# In-Vitro Hemocompatibility of Polyaniline Functionalized by Bioactive Molecules

**DOI:** 10.3390/polym11111861

**Published:** 2019-11-11

**Authors:** Kateřina Skopalová, Zdenka Capáková, Patrycja Bober, Jana Pelková, Jaroslav Stejskal, Věra Kašpárková, Marián Lehocký, Ita Junkar, Miran Mozetič, Petr Humpolíček

**Affiliations:** 1 Centre of Polymer Systems, Tomas Bata University in Zlín, 760 01 Zlín, Czech Republic; skopalova@utb.cz (K.S.); capakova@utb.cz (Z.C.); vkasparkova@utb.cz (V.K.); lehocky@utb.cz (M.L.); 2 Institute of Macromolecular Chemistry, Academy of Sciences of the Czech Republic, 162 06 Prague 6, Czech Republic; bober@imc.cas.cz (P.B.); stejskal@imc.cas.cz (J.S.); 3 Department of Hematology, Tomas Bata Regional Hospital in Zlín, 762 75 Zlín, Czech Republic; pelkova@fhs.utb.cz; 4 Faculty of Humanities, Tomas Bata University in Zlín, 760 01 Zlín, Czech Republic; 5 Faculty of Technology, Tomas Bata University in Zlín, 760 01 Zlín, Czech Republic; 6 Jožef Stefan Institute, Jamova 39, Ljubljana 1000, Slovenia; ita.junkar@ijs.si (I.J.); miran.mozetic@ijs.si (M.M.)

**Keywords:** polymer conductivity, conducting polymer, polyaniline, hemocompatibility

## Abstract

Hemocompatibility is an essential prerequisite for the application of materials in the field of biomedicine and biosensing. In addition, mixed ionic and electronic conductivity of conducting polymers is an advantageous property for these applications. Heparin-like materials containing sulfate, sulfamic, and carboxylic groups may have an anticoagulation effect. Therefore, sodium dodecylbenzenesulfonate, 2-aminoethane-1-sulfonic acid and *N*-(2-acetamido)-2-aminoethanesulfonic acid were used for modification of the representative of conducting polymers, polyaniline, and the resulting products were studied in the context of interactions with human blood. The anticoagulation activity was then correlated to surface energy and conductivity of the materials. Results show that anticoagulation activity is highly affected by the presence of suitable functional groups originating from the used heparin-like substances, and by the properties of polyaniline polymer itself.

## 1. Introduction

The hemocompatibility of any material is a crucial factor in its application in biomedicine, regenerative medicine, or biosensors. The phenomenon of hemocompatibility is very complex and comprises various processes of which blood coagulation remains at the center of attention when tissue engineering and biosensing applications are considered. Any material that comes in contact with blood should not induce coagulation either by disrupting physiological blood flow or by interactions of the blood with the surface of a material on a molecular level.

In some special applications (e.g., biosensing or tissue engineering of electrosensitive tissues) the conductivity of materials can be an advantage. However, common conducting materials (such as metals or metal oxides) can induce an undesired reaction on contact with tissues (e.g., because of their different elasticity compared to native tissue). Therefore, conducting polymers can be more appropriate. Moreover, conducting polymers have a huge potential for the mentioned applications as they show mixed ionic and electronic conductivity, which is preferred for electrically active biointerfaces. The effect of conductivity on the biological systems is a comprehensive issue. We, therefore, refer to the publication of Rivnay et al. [1], who discuss the topic of ionic and electronic conductivity in depth.

Polypyrrole (PPy), poly(3,4-ethylenedioxythiophene) (PEDOT) and polyaniline (PANI) can be considered as the most frequently studied conducting polymers. The biocompatibility of these polymers was recently studied in many research works. For example, the Ramanaviciene et al. [2] tested the effect of PPy particles on mouse peritoneum cells. The action of injected PPy particles was dependent on their concentration and duration of treatment. Nevertheless, the amount of neutrophils did not exceed the limit values at any of the particle concentrations used. Vaitkuviene et al. [3] also investigated the effect of PPy nanoparticle concentration on living systems. It has been demonstrated that PPy nanoparticles exhibit a cytotoxic effect on murine embryonic stem cells, murine hepatoma cells, and human Jurkat T lymphocytes at concentrations higher than 19.4 µg·mL^−1^. From a practical point of view, the polymer films are more important as regards bio applications. Vaitkuviene et al. [4] coated gold-plated glass slides by PPy. Proliferation of mouse stem cells on the treated surfaces was comparable to reference (tissue plastic). The PANI, which is the object of this work, was also previously studied in the context of biocompatibility. Its biocompatibility and especially the cytotoxicity were tested on pristine PANI salt and base [5], as well as on its globular and nanotubular forms [6]. In the scientific literature, it is often mentioned that PPy exhibits favorable properties compared to PANI. However, the study of Humpolíček et al. [7] dealing with the comparison of the biocompatibility of PANI and PPy in terms of cytotoxicity and embryotoxicity showed that the form of polymer (salt vs. base) is more important than its type (PPy vs. PANI). Likewise, the present study compares, inter alia, two forms of PANI, salt and base.

The amounts of information found in the scientific literature about the hemocompatibility of conducting polymer differ. Whilst PPy has been intensely studied, predominantly in functionalized forms or as a part of composites, PEDOT or polyaniline has been investigated as possible biomaterials but their hemocompatibility has not been studied. Li et al. [8] dealt with the blood compatibility of PPy films functionalized by heparin. The compatibility was evaluated using plasma re-calcification time and platelet adhesion. The results showed that, with immobilized heparin, platelet adhesion and platelet activation on PPy films were significantly suppressed and, moreover, the plasma re-calcification time was considerably prolonged.

Ferraz et al. [9] tested the hemocompatibility of nanocellulose/PPy membranes modified by a stable heparin coating. The results demonstrated that the heparinized composites were comparable with hemocompatible polysulfone regarding platelet adhesion and thrombin generation, whereas, in terms of complement activation, they were more biocompatible than commercially available membranes. Mao et al. [10] investigated *O*-butyryl chitosan-grafted PPy film for blood compatibility using platelet-rich plasma. The surface with immobilized *O*-butyryl chitosan exhibited lower platelet adhesion and fibrinogen adsorption compared to unmodified PPy. The films showed good blood compatibility and high electrical conductivity.

Previously it was mentioned that the functionalization of neat PANI films with poly(2-acrylamido-2-methyl-1-propanesulfonic acid) (PAMPSA) results in the hindering of blood coagulation [11]. Humpolíček et al. [11] followed up the studies of Paneva et al. [12] and Yancheva et al. [13] who have shown that PAMPSA can act against blood clotting in a similar way as heparin, either alone or incorporated in copolymers. The biological activity of PAMPSA was studied by Šorm et al. [14] who found out that some methacrylic copolymers that contain (similarly to heparin) sulfate, sulfamic, and carboxylic groups have an anticoagulation effect. Therefore, the compounds holding some of these functional groups were chosen for the functionalization of neat PANI films in this work. Dodecylbenzenesulfonic acid sodium salt (SDBS), 2-aminoethane-1-sulfonic acid (taurine), and *N*-(2-acetamido)-2-aminoethanesulfonic acid (ACES) were, therefore, chosen as modifiers with a potential anticoagulation effect.

## 2. Materials and Methods

### 2.1. Sample Preparation

**Figure 1:**
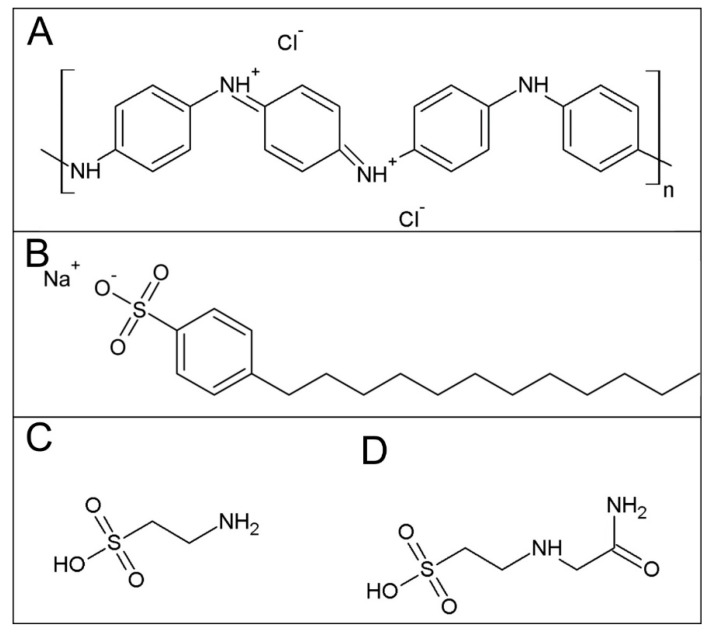
The formula of (**A**) PANI hydrochloride, (**B**) SDBS, (**C**), taurine, and (**D**) ACES.

PANI films containing the above-given compounds (Figure 1) were prepared using two different procedures: (1) By modification of the surfaces of neat PANI films, PANI salt, or PANI base with SDBS, taurine or ACES, and (2) by adding the respective substance directly into a reaction mixture of aniline hydrochloride and ammonium peroxydisulfate used for the preparation of PANI.

Neat PANI films were prepared according to a standard procedure described by Stejskal and Gilbert [15]. Aniline hydrochloride (2.59 g, Penta, Czech Republic) was dissolved in water to a 50 mL solution, and ammonium peroxydisulfate (5.71 g, Lach-Ner, Czech Republic) was similarly dissolved to a 50 mL solution. Both solutions were mixed and immediately poured onto the polypropylene foil and into polyethylene terephthalate (PET) blood-collection tubes. After 1 h, the reaction mixture was removed and the resulting films of green conducting salt (PANI-S) deposited on the foils/walls were rinsed with 0.2 M hydrochloric acid, followed by methanol, and were left to dry in air. Some of the films were deprotonated by immersion in 1 M ammonium hydroxide for 12 h and thus converted to blue, non-conducting films of PANI base (PANI-B). In order to prepare reprotonated films, the samples of neat PANI-B were exposed to 2 wt % solutions of SDBS, taurine or ACES (all from Sigma-Aldrich, St. Louis, MO, USA). Please note that the reprotonation of PANI-B with the individual compounds manifests itself by the reverse change in color from blue to green. The process was slow and took several weeks as a rule (for reprotonation with ACES it took three months). Then, the solutions were removed, and the films were rinsed with methanol and left to dry in air. Modified PANI-S films were prepared by simple pouring each of the 2% solutions of SDBS, taurine or ACES onto the neat film. After 24 h, the solutions of bioactive substances were removed, and the films rinsed with methanol and dried in air. The samples were labelled as PANI-BSDBS, PANI-BTaurine, PANI-BACES, PANI-SSDBS, PANI-STaurine, and PANI-SACES.

The second type of PANI film was prepared by adding the respective substance into a mixture of aniline hydrochloride and ammonium peroxydisulfate. Aniline hydrochloride (2.59 g) was again dissolved in 50 mL of an aqueous solution of SDBS or taurine (40 g·L^−1^). Then, the aqueous solution (50 mL) of ammonium peroxydisulfate (5.71 g) was added, and the reaction mixture was stirred and poured over the substrates (polypropylene foil and blood-collection tubes). The reaction was left to continue for 1 h. The films of PANI salts formed on the substrates containing respective dopants were then rinsed with 0.2 M HCl and were left to dry in the air. The samples were labelled as PANI-MSDBS and PANI-MTaurine. Polyaniline powders obtained after polymerization along with the films were treated similarly and used for conductivity determination.

### 2.2. Surface Energy Measurement

Contact angle data was obtained with a Surface Energy Evaluation System (Advex Instruments, Brno, Czech Republic). Deionized water, ethylene glycol, and diiodomethane were utilized as test liquids. The volume of droplets was set to 5 µL for all experiments to avoid errors connected with gravity acting on the sessile drop. Five contact angle readings were averaged to obtain one representative value. The free energy of the film surfaces was determined by the Lifshitz–van der Waals “acid–base” model. Total surface energy (γ^tot^) was calculated and reported.

### 2.3. Conductivity

The conductivity of the polyaniline powders collected after polymerization and compressed to pellets was measured by the four-point van der Pauw method. A programmable electrometer with an SMU Keithley 237 current source and a Multimeter Keithley 2010 voltmeter (Keithley instrument, Cleveland, OH, USA) with a 2000 SCAN 10-channel scanner card were employed. Measurements were carried out at ambient temperature.

### 2.4. Anticoagulation Test

In all tests, venous blood was collected from healthy donors by venipuncture using the vacuum blood collection system into the 5 mL collecting tubes (VACUETTE, Greiner Bio-One, Kremsmünster, Austria) after obtaining informed consent. All tests were conducted in accordance with the Helsinki Declaration. Plasma was prepared from the venous blood by centrifugation (15 min, 3000× *g*). Plasma was subsequently transferred to the blood-collection tubes coated with studied PANI films. The measurement of coagulation was performed using an instrument commercially used in hospitals, the SYSMEX CA-1500 (Siemens, Erlangen, Germany). The principle of measurement is based on the change of turbidity (measured at a wavelength of 660 nm) as a consequence of adding coagulation reagent which induces the formation of fibrin clothes. In the present study, the following coagulation parameters in human blood plasma treated with 0.109 M citric were studied: (1) Thrombin clotting time (TCT), (2) activated partial thromboplastin time (aPTT), and (3) prothrombin time (PT).

## 3. Results and Discussion

Hemocompatibility is a complex process and its testing by common in-vitro methods is not able to comprehensively cover all its aspects. Blood coagulation as a whole includes not only the coagulation cascade but also the platelet adhesion and can be, for example, triggered by various biochemical cues or blood rheology (flow velocity, flow turbulence, shear stress) [16]. Especially in the case of platelet adhesion, the shear rates of the fluid are important. In this work, we focused on one of the important aspects of blood coagulation, namely on the detection of coagulation induced by the surface properties of modified PANI films. Although the biological properties of PANI in its native or modified form have already been studied, and cytotoxicity [5], interaction with stem cells [17], or interaction with tissues have already been reported, another important parameter of biocompatibility, namely interaction with blood has only been the subject of a few studies. The effect of PANI itself on platelet adhesion, hemolysis and plasma recalcification time has been studied by Li et al. [18]. Here, PANI-coated polyurethane (PU) fibers were tested. Platelets on PANI-coated PU demonstrated lower aggregation (6.87 × 10^5^ cm^−2^) than platelets on PU fibers without modification (15.63 × 10^5^ cm^−2^). As regards hemolysis, according to requirements of ISO 10993-4, materials with hemolysis values <5% are considered safe in contact with blood. In the study, PANI-PU fibers exhibited hemolysis values of 0.14% and non-coated PU fibers 0.21%. The tests also showed that PANI prolonged plasma recalcification time by 13 s, improving thus anticoagulation. The impact of PANI on the blood coagulation can be also improved by modification of its surface by bioactive substances. For example, a work of Zhang et al. [19] studied modifications of PANI films with poly(ethylene glycol) (PEG) to reduce the adhesion of proteins and platelets on its surface. In this case, the PEG chain was covalently bound to PANI film. Adhesion of bovine serum albumin (BSA) and γ-globulin decreased with increasing concentration of surface-bound PEG. The same results were obtained for platelet adhesion. Li and Ruckenstein [20] tested BSA and platelet adhesions on native PANI and poly(ethylene oxide) (PEO) grafted films. Correspondingly to the previous study, modification of the PANI film with hydrophilic PEO also reduced protein adhesion, in this case by 80%. Platelet adhesion also decreased significantly. In the case of the unmodified film, there was adhesion of 18 × 10^3^ mm^−2^. For the modified film, the adhesion value was 2.1 × 10^3^ mm^−2^. Similar results were achieved in work of Humpolicek et al. [11]. This report investigated the impact of PANI functionalized with PAMPSA on blood coagulation and found out that PANI films reprotonated with this high-molecular-weight acid hindered blood coagulation by interaction with three of the coagulation factors: Xa, Va, and IIa. Moreover, PAMPSA-modified PANI also reduced platelet adhesion. It was generally expected that methacrylic copolymers containing similarly to heparin the sulfate, sulfamic, and carboxylic groups, may have an anticoagulation effect. With respect to this assumption, SDBS, taurine, and ACES might have the potential to influence blood compatibility as well. As PANI is a versatile material that can easily be modified by a variety of methods, the PANI films were prepared using the above-mentioned substances either by their direct addition into the reaction mixture with subsequent preparation of films, or by modification of neat PANI-S and PANI-B films via pouring over the solutions of each of these substances.

The standard polyaniline salt, PANI-S, contains chloride counter-ions (Figure 1A), as well as sulfate ions originating from the reduction of peroxydisulfate. These counter-ions are removed after the conversion to PANI-B. After immersion of PANI-B into the solution of, e.g., taurine, immobilization of this substance onto PANI film takes place resulting in their mutual salt (PANI-BTaurine) containing corresponding taurine counter-ions. As regards PANI-S films modified with studied heparin-like substances, it can be assumed that a layer of the respective substance remains on the surface of neat PANI. When PANI is prepared in the presence of taurine in reaction mixture, PANI-MTaurine, the films contain chloride, sulfate, and taurine counter-ions. The PANI films prepared in various ways thus differ in the type and occurrence of counter-ions, or more general in surface chemistry.

Blood coagulation can be triggered by contact with interfaces due to the following three crucial characteristics/properties of a surface: (1) The surface energy, (2) the acidity (pH), and (3) the interaction of surfaces with coagulation factors of blood. The adherence of plasma proteins and the subsequent adherence of platelets and leukocytes are also of importance regarding the plasmatic coagulation, which again depends on the surface characteristics of the material. The surface energies measured on modified PANI films were similar for almost all samples (Table 1) and did not differ from γ^tot^ determined on neat PANI-S and PANI-B films [21]. The exception here was PANI-BACES with γ^tot^ equal to 41 ± 1 mN cm^−1^. For practical application, the stability of the samples is crucial, and the effect of aging on the surface energy was also determined. The surface energy of all samples was stable for at least two weeks, except for the PANI-BTaurine and PANI-STaurine films (Table 1).

It is known that the physiological pH of blood lies within a tight range from 7.31 to 7.42 [22], and several non-physiological reactions can occur as a consequence of the pH changes, including inhibition and prolongation of coagulation processes. The pH of blood after being in contact with the samples was therefore determined, and it was unambiguously confirmed that the pH was not notably influenced by any of the tested samples.

Conductivity is another important characteristic of conducting polymers. The standard films of PANI-S commonly show conductivity within units of S·cm^−1^ [23]. The conductivity measurements on fresh samples, and after 7 and 14 days of their storage at room temperature are summarized in Table 2. A certain conductivity drift is likely to be associated with still decreasing sample humidity. Measurements revealed that only the samples synthesized with SDBS or taurine present in the reaction mixture showed reasonably high conductivity and all other films exhibited conductivity lower than 10^−4^ S·cm^−1^. It is beyond the scope of this manuscript to define the relationship between the conductivity of materials and their applicability in biomedicine. This is a comprehensive issue and the situation is complicated not only by the fact that conducting polymers combine ionic and electronic conductivity but also by the different resistivity of various tissues (e.g., from 100 Ω·cm for blood to 1000 Ω·cm for some tissues) [24] which corresponds to the conductivity of 0.01 to 0.001 S·cm^−1^. In this context, the conductivity of here-tested PANI samples, especially these where taurine was present in the reaction mixture, is significantly higher reaching ~18 S·cm^−1^.

The coagulation parameters of the blood after contact with the studied surfaces are presented in Table 3. The thrombin clotting time (TCT), activated partial thromboplastin time (aPTT), and prothrombin time (PT) were studied as clinically relevant parameters using the methods described earlier [11]. Similarly to neat PANI-S and PANI-B [11], none of the PANI modified with SDBS, taurine or ACES significantly impacted blood coagulation (Table 3), and all the coagulation parameters were within their respective physiological ranges. The expected impact of taurine, SDBS, or ACES on the hemocompatibility was not hence confirmed. So far, the only compound suitable for influencing the hemocompatibility of PANI is, as previously described [11], PAMPSA. Here, an interesting question arises with regard to the reason why SDBS, taurine, or ACES were not able to modify the hemocompatibility of PANI even though they contain functional groups similar to PAMPSA.

Based on the results presented in Table 1, Table 2 and Table 3 and on previously published findings, the reason for this can be found in the low-molecular-weight character of these substances, which in all three cases lies below 350 g·mol^−1^. On the other hand, in the case of hemocompatible PANI-PAMPSA, its anticoagulation activity can be also assigned to the presence of the high-molecular-weight PAMPSA polyanion (molar mass ≈ 10^6^ g·mol^−1^) bound to polycationic PANI surface. During reprotonation, a fraction of anions on a PAMPSA chain is bound as counter-ions to the PANI backbone. However, a substantial fraction of the functional sulfo groups still remains free to interact with blood and can act as an anticoagulant agent. In this context, it can be mentioned that also the best-known anticoagulation agent, heparin, is a polymer. This polysaccharide consists of chains containing 1,4-bonded residues of uronic acid and D-glucosamine, and its molar mass ranges from 3000 to 30,000 g·mol^−1^, with an average of 15,000 g·mol^−1^ [25]. However, its anticoagulant activity is closely related to a unique pentasaccharide sequence necessary for linking to antithrombin [26]. In the heparin chain without this pentasaccharide sequence, the anticoagulation activity is absent [27].

In summary, the present pilot study covers only a limited part of the complex hemocompatibility process. The impact of above-mentioned parameters, such as blood flow velocity, flow turbulence, shear stress, or tension on the hemocompatibility of PANI-based surfaces has not been studied so far. It, therefore, opens the possibility for further testing of the effect of the low-molecular-weight substances with sulfo groups on the hemocompatibility, which can be conducted under physiological flow conditions and using blood flow models.

## 4. Conclusions

The PANI films modified by substances with anticipated anticoagulant activity, sodium dodecylbenzenesulfonate (SDBS), 2-aminoethane-1-sulfonic acid (taurine) and *N*-(2-acetamido)-2-aminoethanesulfonic acid (ACES) have been studied. The hemocompatibility tests conducted on these PANI films confirmed that none of them showed anticipated anticoagulation activity, though the functional groups typical for anticoagulation substances were present. The absence of the activity can be ascribed to the low molecular weight of these compounds used for PANI modification. It can be, therefore, concluded that, in addition to the presence of suitable functional groups in the molecule, the dopants introducing anticoagulation activity should exhibit polymer-like character with sufficiently high molecular weight.

## Figures and Tables

**Table 1:** The surface energy of modified PANI surfaces, γ^tot^, determined immediately after preparation, and after 7 and 14 days on the stored under laboratory conditions. The values are compared with γ^tot^ of neat PANI-S and PANI-B films.

		γ^tot^ ± SD (mN·m^−1^)	
	Day 0	Day 7	Day 14
PANI-BSDBS	51 ± 3	51 ± 2	50 ± 7
PANI-SSDBS	51 ± 3	47 ± 4	44 ± 4
PANI-BTaurine	51 ± 7	53 ± 2	38 ± 12
PANI-STaurine	55 ± 8	45 ± 7	47 ± 2
PANI-BACES	41 ± 1	37 ± 1	45 ± 0
PANI-MSDBS	46 ± 5	47 ± 4	46 ± 4
PANI-MTaurine	53 ± 4	44 ± 10	46 ± 4
PANI-S ^(a)^	52.54	n.d.	n.d.
PANI-B ^(a)^	50.88	n.d.	n.d.

^(a)^ Reproduced from [21]; n.d. not determined.

**Table 2:** The conductivity of bulk PANI (S cm^−1^) containing the studied substances.

Sample	Day 0	Day 7	Day 14
PANI-MSDBS	7.0 ± 0.03	5.7 ± 0.002	5.2 ± 0.004
PANI-MTaurine	17.9 ± 0.01	16.5 ± 0.004	15.8 ± 0.003

**Table 3:** Impact of PANI with modified surfaces on selected coagulation parameters, prothrombin time (PT), activated partial thromboplastin time (aPPT), thrombin clotting time (TCT), expressed as times to the coagulation start.

	PT [s]	aPPT [s]	TCT [s]
Reference	12.1 ± 0.1	25.2 ± 0.5	16.7 ± 0.3
PANI-BSDBS	12.2 ± 0.6	29.5 ± 4.7	20.0 ± 2.2
PANI-SSDBS	11.8 ± 0.2	25.6 ± 0.3	18.3 ± 0.3
PANI-BTaurin	11.8 ± 0.0	25.4 ± 0.5	17.7 ± 0.5
PANI-STaurin	11.8 ± 0.2	26.9 ± 0.6	19.2 ± 0.4
PANI-BACES	12.3 ± 0.0	25.4 ± 0.3	15.4 ± 0.4
PANI-MSDBS	12.0 ± 0.1	25.8 ± 0.6	18.2 ± 0.5
PANI-MTaurin	12.1 ± 0.3	30.0 ± 2.5	19.7 ± 0.6

Note: As reference coagulation parameters of donor blood were used. Normal ranges for coagulation parameters of a healthy person: PT 11.0–13.5, aPTT 25–32, TCT below 20 s. The values are expressed as mean value ± standard deviation of three tests.
